# Demographic, Premorbid, and Clinical Characteristics of Schizophrenia Spectrum Patients with High and Low Polygenic Liability to the Disorder

**DOI:** 10.3390/diseases13030066

**Published:** 2025-02-21

**Authors:** Margarita Alfimova, Marina Gabaeva, Tatyana Lezheiko, Victoria Plakunova, Yulia Chaika, Vera Golimbet

**Affiliations:** Mental Health Research Center, 115522 Moscow, Russia; gabaeva@yandex.ru (M.G.); lezheiko@list.ru (T.L.); golimbet@mail.ru (V.G.)

**Keywords:** schizophrenia, polygenic risk scores, risk factors, premorbid functioning, PANSS

## Abstract

Background/Objectives: Schizophrenia is a clinically heterogeneous complex disorder with a substantial polygenic basis. The discovery of phenotypes indexing genetic differences advances research into the schizophrenia etiology but has proven to be challenging. The study aimed to further clarify the relationships of schizophrenia polygenic risk scores (SZ-PRSs) with a comprehensive array of schizophrenia antecedents and presentations using a culturally and ethnically homogeneous sample of schizophrenia spectrum patients. Methods: The top and bottom deciles (*n* = 172) of the SZ-PRS distribution in a group of 861 patients were compared on information derived from medical records using logistic regression. Results: High SZ-PRSs were associated with female sex, family history of a wide range of neuropsychiatric conditions, moderately poor premorbid social and cognitive adjustment in childhood, the schizophrenia diagnosis, and positive and “abnormal” psychomotor symptoms. The low-SZ-PRS group demonstrated an accumulation of both individuals with milder forms of SZ spectrum disorders and those with severe premorbid abnormalities in the social, cognitive, and neurological domains. Conclusions: The results highlight moderately poor premorbid social and cognitive adjustment as characteristic manifestations of the polygenic component of the schizophrenia etiology and provide the first piece of PRS-based evidence for the long-standing idea of a higher liability threshold in women. The presence of milder and severe cases in the bottom SZ-PRS decile, suggesting its etiological heterogeneity, might be an important source of the inconsistency in the previous research on SZ-PRSs’ relationship with schizophrenia phenotypes and should be considered in future studies.

## 1. Introduction

Schizophrenia (SZ) is a clinically heterogeneous disease, with significant contribution of genetic and environmental factors to its etiology. The genetic factors include both a polygenic component, which comprises many common genetic risk variants with a small effect, and more penetrant rare and new single-nucleotide variants and copy number variations [1]. The discovery of phenotypes indexing genetic differences between patients would significantly advance research into the SZ etiology and the development of personalized psychiatry [2,3]. To date, family and molecular genetic studies have revealed some links between genetic burden for SZ and SZ-related phenotypes, with strong associations being found only for the severity of negative symptoms [3]. Data on other characteristics remain inconsistent, and many clinically important phenotypes have been investigated in isolation or in single studies, or have been missed. Thus, further research is needed to clarify the relationship between genetic factors and SZ antecedents and presentation.

The main tool to explore the influence of common genetic variants on clinical heterogeneity is polygenic risk scores (SZ-PRSs), which are a sum of SZ risk alleles weighted by the allele effect size derived from genome-wide association studies (GWASs). In most PRS-based investigations, correlations with quantitative clinical phenotypes have been evaluated, with mixed findings [3,4,5,6]. Several recent studies have explored top-down clustering of patients based on deep clinical phenotyping [7,8,9] or bottom-up clustering based on PRSs for SZ and related disorders [10]. Their results suggest that leveraging genetic heterogeneity that underlies clinical heterogeneity by comparing homogeneous groups of patients could be fruitful.

Here, we used the extreme group approach [11], a cost-efficient alternative to bottom-up clustering, to analyze the relationship between SZ-PRSs and a comprehensive array of SZ-related phenotypes in patients with SZ spectrum diagnoses. We compared the top (TD) and bottom deciles (BD) of the SZ-PRS distribution on these phenotypes, assuming that the group with the highest SZ-PRS is relatively etiologically homogeneous, since this group does not include phenocopies and most of the genetic components underlying different symptoms present in the patient’s genome, while the group with low SZ-PRSs is presumably more diverse, in accordance with the classical multifactorial threshold model of SZ transmission [12], in which one would expect a greater contribution from both non-genetic and rare genetic factors to the SZ development in the absence of substantial polygenic liability.

The study aimed to confirm the PRS- and family-based data on the associations of genetic burden for SZ with the disease phenotypic variability and to expand them regarding poorly investigated environmental and premorbid characteristics associated with the disease [13], using a culturally and ethnically homogeneous population and SZ-PRSs derived from the largest SZ GWAS to date [14]. Built on the conclusions of a recent systematic review [3], we expected that, compared to the SZ-PRS BD, the SZ-PRS TD would be characterized by a larger proportion of women, higher family history of severe mental illness, earlier illness onset, higher rate or severity of negative symptoms, and poorer functioning. In addition, although the PRS-based data regarding the respective phenotypes are less consistent or absent, we hypothesized that the TD would have a poorer premorbid adjustment and higher lifetime rates of suicidality and addictions. Finally, we speculated that the TD would be associated with fewer environmental risk factors, since the manifestation of SZ in this group might not require an external trigger.

## 2. Materials and Methods

### 2.1. Sample

The original sample consisted of 861 unrelated Russian individuals with SZ spectrum diagnoses based on the International Classification of Diseases (ICD-10) criteria for categories from the F2 chapter (49% women; mean age 28.68 ± 12.85 years, aged 5–69 years; diagnoses were as follows: F20–82%, F25–9%, F23–1%, F21–8%). The sample was extracted from the database of the Clinical Genetics Laboratory of the Mental Health Research Center (MHRC, Moscow) described earlier [15] and comprised patients for whom data for the SZ-PRS calculation were available. Notably, all participants were inpatients of two local hospitals. The TD (*n* = 86) and BD (*n* = 86) groups were composed of patients whose SZ-PRSs belonged to the top and bottom deciles of the SZ-PRS distribution in the original sample. The study has been performed in accordance with the ethical standards laid down in the 1964 Declaration of Helsinki and its later amendments and the national law “On psychiatric care and guarantees of the rights of citizens during its provision”. Approval was granted by the Ethics Committee of the MHRC (Date 18.03.2022/No. 873). According to national law, all participants aged 15 or older provided written informed consent to participate in the study; for each underage participant, written informed consent was obtained from a legal representative. The study did not include any vulnerable patients other than underage participants.

### 2.2. Genotyping and SZ-PRS Calculation

Genomic DNA was extracted from blood samples. Genotyping was made within different research projects using HAP610-QUAD, PMDA, PsychArray, and GSA v2/v3 BeadChips. Genetic data processing and quality control were carried out with PLINK v.1.9, according to the standard pipeline [16]. The filtering was based on the missingness rate of single-nucleotide polymorphisms (SNPs) (>1%) and individuals (>5%), Hardy–Wienberg equilibrium violation (*p* < 10^−6^), autosomal heterozygosity deviation (>±3 standard deviations (SDs) from the samples’ heterozygosity rate mean), database/genetic sex mismatch, relatedness (pi-hat > 0.2), and population structure checking, with outliers from the study ethnic group being excluded based on the principal component (PC) analysis. Data were imputed by the Minimac4 tool with the HRC r1.1 using the European panel. Ambiguously oriented SNPs were excluded. PRSs were calculated using 643,063 SNPs and their corresponding weights from the PGC3 combined ancestry GWAS [14] by means of the LDpred2-auto tool [17] available in the R package “bigsnpr” (v1.12.2), according to the author’s guidelines (https://privefl.github.io/bigsnpr/articles/LDpred2.html (accessed on 1 November 2023)). A sample of healthy unrelated Russian individuals (controls, *n* = 760) from the same database, who had no first- or second-degree relatives with SZ or affective spectrum disorders, served as the LD reference. The SZ-PRSs were standardized using the controls’ mean and SD. Ancestry-related PCs were calculated with PLINK (-pca command).

### 2.3. Phenotyping

Phenotypic data were extracted from patients’ medical records and partly verified using a predefined set of questions given to the patient by a research team member at the time of recruitment. We obtained information on the following factors: date (season of birth, SOB) and place of birth (urbanicity); sex; family history of psychiatric illnesses and a wider spectrum of neuropsychiatric conditions; number of siblings; birth order; maximum educational attainment; marital history; employment; obstetric complications (OCs); adverse childhood experiences (ACEs); premorbid neurological and neurodevelopmental conditions; premorbid somatic, cognitive, and social functioning; illicit drug use; age at onset and at first hospitalization; the latest ICD-10 diagnosis; lifetime ICD-10/11 SZ symptom domains, including three psychomotor/catatonic domains [18] and excluding depressive and manic mood states; history of suicidal ideation and attempts; and lifetime psychoactive substances misuse. A five-factor Positive and Negative Syndrome Scale (PANSS) model with a built-in two-factor model of the negative syndrome [19] was used to compare the groups in terms of symptom severity at the time of recruitment. Full definitions of all demographic, environmental, premorbid, and clinical characteristics are provided in the Supplementary Material, Table S1.

### 2.4. Data Analysis

At the first stage, frequencies of categorical phenotypes were analyzed. Due to missing data and age constraints for some variables (e.g., marital history was assessed in patients 18 years or older), the proportion of patients included in each PRS–phenotype association varied but was not less than 50% in each decile group (see Table S1 for the exact number of participants in each analysis). No imputation of missing data was applied. Phenotypes equal in more than 90% of the sample were excluded from subsequent analysis. No preliminary assessment of the potential interactions between genetic and environmental risk factors was carried out. The main analysis was performed by logistic regression using a hierarchical approach. The null model included age, the first two ancestry-related PCs, and the site of genotyping; for symptoms, we also added illness duration. Each time one of the phenotypic characteristics of interest was introduced into the logistic model as a predictor, the significance of model improvement and the increment of explained variance relative to the null model were evaluated, as well as odds ratios (ORs) for the phenotype. To improve the precision of the effect estimates, we also calculated ORs and bias-corrected accelerated 95% confidence intervals (bca 95% CIs) based on up to 500 bootstrap samples. The significance level was set at α = 0.05, and was two-tailed. To control for multiple comparisons, the Benjamini–Hochberg procedure was applied. Given that our analysis was hypothesis-driven, the number of the variables analyzed was <40, and they were interrelated; an FDR-value of <0.10 were considered significant. All statistical procedures were conducted with JASP 0.16.4.0 [20].

## 3. Results

The characteristics of the TD and BD are shown in Table 1; age distribution by group is given in Table S2. The TD had high SZ-PRS values of about three SDs above the controls’ mean, while the BD showed average SZ-PRS values (mostly within one SD below the controls’ mean). The groups were similar in age (t = 0.10, *p* = 0.922) and illness duration (Mann–Whitney U = 3569, *p* = 0.894), which made it possible to compare them in terms of symptom severity at the time of recruitment.

**Figure 1:**
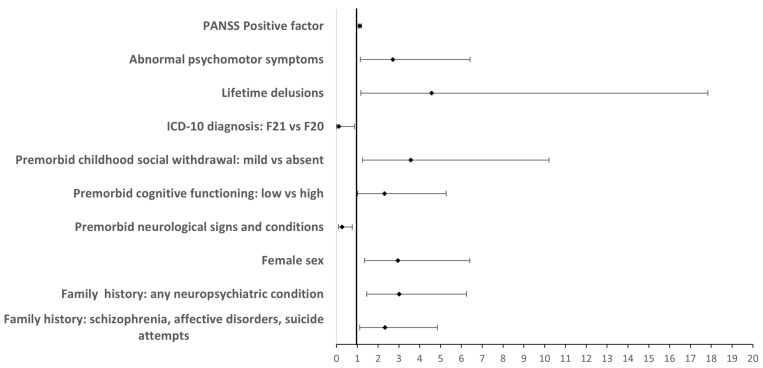
Forest plot of effect sizes for significant predictors of SZ-PRS top-decile membership. Effects (OR, 95% CI) significant at FDR < 0.1 are presented.

After adjusting for multiple comparisons, the TD was associated with female sex, higher family history of severe mental illness, a wider range of neuropsychiatric conditions, lower frequency of premorbid neurological signs and conditions, mild decrease in premorbid cognitive and social functioning, SZ diagnosis (F20), lifetime delusions, and the severity of PANSS positive symptoms and psychomotor symptoms of the abnormal type (Figure 1; Table 2; see Table S3 for the full data). Specifically, regarding premorbid functioning, as compared to the BD, the TD group showed a significant increase in the proportion of patients with below-average academic performance and mild social withdrawal in the form of shyness, isolation, and few or no friends, as well as a trend to a decreased proportion of patients with severe cognitive (i.e., intellectual disability) and social (autistic-like) problems. Some of the associations, however, were not robust since the bca 95% CI of the respective ORs included 1 (Table S3).

Given the influence of sex on a variety of disease characteristics [21,22], we repeated the analysis for all phenotypes, adding sex to the null model. This did not significantly change *p*-values, effect sizes, or ORs (Table S4).

To consider the interrelatedness of the SZ antecedents and presentations and their possible additive effects, we ran two auxiliary analyses. First, we conducted a post hoc stepwise logistic regression adjusted for the confounders to predict decile membership using all 10 significant characteristics simultaneously. The analysis showed that family history of severe mental disorders, family history of a wide spectrum of neuropsychiatric conditions, mild social withdrawal in childhood, and a higher severity of PANSS positive symptoms at the time of recruitment were independent predictors of TD membership at the level of significance or at a trend level (*p* < 0.10) (Table S5). Second, we used the random forest algorithm to classify patients into the TD and BD based on all studied characteristics (Supplementary Material, Forest Analysis Notes, Tables S6 and S7, Figure S1). The analysis yielded unsatisfactory model parameters: out-of-bag accuracy—0.68; AUC—0.58; and F1 score—0.49. As missed data were not imputed, the samples in both analyses were small (*n* = 67 and *n* = 54), and we considered this step as exploratory in nature.

## 4. Discussion

The study confirmed the following hypotheses. First, we found an elevated proportion of female patients in the SZ-PRS TD. One way to account for this result is a female protective effect due to which it may take higher genetic liability for a woman to be diagnosed with schizophrenia and hospitalized than for a man. This explanation has been previously offered for family and epidemiologic data [3]. To the best of our knowledge, our finding is the first piece of PRS-based evidence for the long-standing idea of a higher liability threshold in women. Second, high SZ-PRSs expectedly positively correlated with family history of SZ, affective disorders, and suicide attempts. In addition, we found an accumulation of other neuropsychiatric conditions among close relatives of patents from the TD. These conditions were mainly represented by alcoholism, which was in line with the recently shown correlation between SZ-PRSs and alcohol dependence in individuals without psychotic diagnoses [23] and should be taken into account when considering parental alcoholism as a risk factor for SZ. Third, the TD and BD did differ in premorbid functioning, the pattern of differences being however more complicated than just a poorer premorbid adjustment in the TD. Specifically, the TD tended to comprise fewer individuals with a pronounced cognitive defect/intellectual disability but more patients with moderately low academic achievements than the BD. Similarly, the TD was associated with mild social withdrawal in childhood, while the BD showed a trend towards an increase in severe communication impairments and neurological signs and conditions. Given a higher rate of comorbid intellectual disability in SZ probands carrying rare mutations [24] and the fact that rare genetic disorders frequently involve both neurological and psychiatric phenotypes [25], our results might be considered in favor of the notion of a negative correlation between SZ-PRSs and the presence of rare genetic variants [26,27] and add to the growing literature about the relationships between SZ-PRSs and social and academic premorbid functioning in SZ [28,29,30].

Regarding clinical data, we expected to confirm the correlations of SZ-PRSs with earlier illness onset and negative symptoms [3]. Instead, the deciles differed in the occurrence and severity of the positive domain, specifically delusions and, to a lesser extent, hallucinations, as well as in “abnormal” psychomotor symptoms, mainly mannerisms and posturing, which are a part of the cognitive disorganization cluster [19]. It should be noted that findings regarding the SZ-PRS association with symptoms are inconsistent. While the most powerful correlational study has found an association between SZ-PRSs and the presence and severity of negative symptoms [31], several smaller studies have shown correlations also or exclusively with positive [4,6,32], disorganization [5], or general psychopathological symptoms [33]. Also of note is the association of SZ-PRSs with psychotic symptoms in bipolar disorder [31] and with psychotic experiences and subclinical psychotic symptoms in people without psychosis [32,34]. The inconsistency in the SZ-PRS associations with symptoms could be due to a number of reasons discussed earlier [8,33,35], one of which is not differentiating between primary and secondary negative symptoms. In our study, TD membership was associated with mild social withdrawal in childhood, which might be a precursor of the primary negative symptoms, and as such, this finding could partly support previous data on the associations between negative symptoms and SZ-PRSs.

We were unable to confirm the hypothesis about a decrease in environmental risk factors in the TD. The environmental risk factors were widespread in both groups and could be related to a variety of processes. In people with low SZ-PRSs, they might represent additional exposures, acting through epigenetic processes [36]. In people with high SZ-PRSs, they could be involved in genotype–environment interactions. In the latter case, the presence of genetic risk variants is assumed to provide high sensitivity to specific environmental hazards, as has been recently shown for cannabis use and some aspects of ACEs [37]. Given this, the comparable rate of environmental factors in the TD and BD suggests that targeting modifiable environmental factors could be beneficial even for individuals with a high polygenic liability to SZ. However, not all “environmental” factors might be purely environmental. Specifically, unlike SOB or OCs, which appear to be unrelated to the genetic burden for SZ [38,39], ACEs reflect, to some extent, the genetic component of SZ risk, as the ACE list included such characteristics of a dysfunctional household as parental mental disorders and parental substance misuse [40]. Our sample was not large enough to clarify the relations of ACEs with SZ-PRSs by examining different sets of ACEs, but this should be carried out in future studies.

Finally, in contrast to some previous research [41,42], we did not find a link between SZ-PRSs and substance misuse or suicidality, which might be explained by highly multifactorial etiologies of these phenotypes in SZ [43,44].

Overall, the data on the relationship between SZ-PRSs and different parameters of SZ risk and presentation remain inconsistent. Our findings provide additional support for the association of polygenic predisposition to SZ with family history of psychiatric disorders [45]. At the same time, they do not confirm the data on the association of higher SZ-PRSs with an earlier age of onset [46] or the severity of negative symptoms [3,31]. In contrast to some recent studies [29,30], they imply the role of the SZ-PRS in premorbid academic and social functioning. In addition, we were unable to clarify the complicated issue of the relationship between genetic and environmental factors, for which both independent and synergistic effects on SZ development have been previously found [23,32,37,38,39]. While the overall inconsistency might be partly due to a lack of consensus regarding definitions of phenotypes, the absence of significant differences between the deciles in environmental exposures may also reflect the true complexity of genotype–environment relationships in SZ. All our results, however, should be viewed in light of the study’s limitations.

The main limitation was the way of collecting information. We used all available previously collected data. However, within each project, where patients’ genotypes were determined, different instruments and procedures were applied to gather information, which reduced the reliability of comparing information across patients. Another limitation might be the sample size. It could not be calculated a priori for most factors due to the lack of relevant prior data. However, given the effect sizes found in case–control studies [12], one could expect very small ORs for some factors (e.g., OR for SOB < 1.1). Next, the sample composition should be considered. We included children as young as 5 years old. All children (<13 years old) met diagnostic criteria for SZ, child type (F20.8xx3), from the ICD-10 version adapted in the RF. The diagnoses were made by two psychiatrists from the pediatric unit of the MHRC based on clinical interviews with children and their parents, systematic inpatient observation, and results of physical/neurological examination. The proportions of children did not differ significantly between the TD and BD (about 6% and 7%, respectively; Table S2). We therefore assume that our results are not considerably biased by the inclusion of children. Still, given the lack of international consensus on childhood-onset SZ, the presence of very young patients in the sample may be a source of some heterogeneity. Further, we used an ethnically and culturally homogeneous sample and the most powerful GWAS of schizophrenia to calculate SZ-PRSs, which was a strength of our work. However, the association of SZ-PRSs with individual characteristics and environmental exposures may be influenced by cultural, including religious, factors operating in different ethnic groups. It is conceivable that as more powerful region-specific GWASs of schizophrenia become available, studies of the association between SZ-PRSs and schizophrenia-related phenotypes will be more informative. Finally, not all potential confounders were considered. In particular, each participant received a complex antipsychotic medication regimen. This could affect clinical presentation at the time of recruitment and potentially the course of disease. Unmeasured confounding might also influence the result on sex difference as sex correlates with many biological, lifestyle, and clinical factors.

## 5. Conclusions

Our data suggest that high SZ-PRSs are associated with SZ diagnosis, positive and “abnormal” psychomotor symptoms, and premorbid cognitive and social characteristics such as moderately low academic performance (but not intellectual disability) and social withdrawal in the form of shyness and few friends in childhood. The group with relatively low SZ-PRSs showed a high phenotypic heterogeneity. On the one hand, this group included more people with less severe forms of the disease (i.e., schizotypal disorder) than the high-SZ-PRS group. On the other hand, the low-SZ-PRS group differed from that with high SZ-PRSs with a higher portion of individuals with premorbid neurological symptoms, intellectual disability, and pronounced communication impairments, which was consistent with the notion of an increased number of rare mutations in patients with lower SZ-PRSs [26]. This suggests that a variety of mechanisms might act in the development of SZ spectrum disorders in the case of low polygenic liability, although we did not find an increase in the rate of environmental risk factors in the SZ-PRS BD. The heterogeneity of the lower end of the SZ-PRS distribution might be an important source of the inconsistency in the previous research on the relationship between SZ-PRSs and SZ phenotypes and should be taken into account in future studies.

## Figures and Tables

**Table 1:** Demographic and clinical characteristics of the SZ-PRS top and bottom deciles.

Variable	Top	Bottom
N	86	86
Sex (% women)	60	37 *
Age (years)	27.16 ± 11.87	26.98 ± 12.93
Educational attainment (Median) ^a^	4	5 *
Marital status (% ever been married or had a partner)	25	31
Current education or paid employment (%)	28	37
ICD-10 diagnoses (% F20/F25/F21) ^b^	86/13/1	78/8/14 *
Age at illness onset (years)	17.49 ± 6.36	17.55 ± 7.98
Age at first hospitalization (years)	19.47 ± 7.37	20.41 ± 8.79
Years of illness duration, median (min–max)	5 (0–44)	6 (0–45)
SZ-PRS, median (min–max)	3.00 (2.62–4.28)	−0.59 (−1.71–0.11)

Note. ^a^ According to the International Standard Classification of Education—2011, educational levels 4 and 5 correspond to post-secondary non-tertiary (e.g., higher technical education or training systems) and short-cycle tertiary (i.e., 1–3 years at university) education, respectively. ^b^ At the time of recruitment, two patients with an F21 diagnosis from the BD group had a comorbid diagnosis of F34 (persistent mood [affective] disorders). * *p* < 0.05.

**Table 2:** Significant predictors of SZ-PRS top-decile membership.

Phenotype	% of Patients with the Phenotype		Logistic Regression Parameters (Improvement Compared to the Null Model)
	Top	Bottom	
Family history of SZ, affective disorders, or suicide attempts	46.1	28.8	χ^2^ = 5.27; *p* = 0.022/p_BH_ = 0.081; R^2^_N_ = 0.05
Family history of any neuropsychiatric condition	67.1	41.3	χ^2^ = 9.33; *p* = 0.002/p_BH_ = 0.037; R^2^_N_ = 0.08
Female sex	60.5	37.2	χ^2^ = 7.75; *p* = 0.005/p_BH_ = 0.046; R^2^_N_ = 0.06
Premorbid neurological signs and conditions	16.7	31.1	χ^2^ = 6.79; *p* = 0.009/p_BH_ = 0.067; R^2^_N_ = 0.08
Premorbid cognitive functioning:			χ^2^ = 11.22; *p* = 0.011/p_BH_ = 0.068; R^2^_N_ = 0.10
high	42.0	53.9	
low	44.9	22.4	
very low	10.2	9.2	
intellectual disability	2.9	14.5	
Premorbid childhood social withdrawal:			χ^2^ = 14.46; *p* < 0.001/p_BH_ < 0.037; R^2^_N_ = 0.21
absent	38.6	53.1	
mild	56.8	26.5	
severe	4.6	20.4	
ICD-10 diagnosis:			χ^2^ = 8.37; *p* = 0.015/p_BH_ = 0.074; R^2^_N_ = 0.07
F21	1.2	14.0	
F25	12.8	8.1	
F20	86.0	77.9	
Lifetime delusions	96.1	76.4	χ^2^ = 5.84; *p* = 0.016/p_BH_ = 0.074; R^2^_N_ = 0.06
Psychomotor symptoms—Abnormal type	39.7	20.8	χ^2^ = 5.39; *p* = 0.020/p_BH_ = 0.081; R^2^_N_ = 0.05
PANSS positive factor (scores, M ± SD)	23.86± 5.96	20.21± 6.14	Wald Stat = 8.98; *p* = 0.003/p_BH_ = 0.037

Note. p_BH_—Benjamini–Hochberg-corrected *p*-values.

## Data Availability

The data that support the findings of this study are not publicly available due to the fact that they contain information that could compromise the privacy of the research participants, but they can be made available by the corresponding author, M.A., upon reasonable request.

## Supplementary Materials

The following supporting information can be downloaded at https://www.mdpi.com/article/10.3390/diseases13030066/s1, Table S1: Definitions and coding of phenotypes and the number of patients included in the respective analysis; Table S2: Age distribution by group; Table S3: Phenotypic differences between the top and bottom deciles according to logistic regression; Table S4: Phenotypic differences between the top and bottom deciles according to logistic regression with sex as an additional covariate; Table S5. Results of the stepwise logistic regression to predict top-decile membership (vs. bottom-decile membership) based on statistically significant phenotypes from Table S3; random forest analysis notes; Table S6: Evaluation metrics; Table S7: Feature importance; Figure S1: ROC curve plots.

## Abbreviations

The following abbreviations are used in this manuscript:

SZ	Schizophrenia
SZ-PRS	Schizophrenia polygenic risk score
GWAS	Genome-wide association study
TD	Top decile
BD	Bottom decile
PGC	Psychiatric Genomics Consortium
ICD	International Classification of Diseases
SNP	Single-nucleotide polymorphism
PC	Principal component
SOB	Season of birth
OC	Obstetric complication
ACE	Adverse childhood experience
PANSS	Positive and Negative Syndrome Scale
OR	Odds ratio
bca CIs	Bias-corrected accelerated confidence intervals
