# Supplementary material for: Demographic, Premorbid, and Clinical Characteristics of Schizophrenia Spectrum Patients with High and Low Polygenic Liability to the Disorder
Source: Diseases. 2025 Feb 21;13(3):66. doi: 10.3390/diseases13030066 (PMC11941017; doi:10.3390/diseases13030066)

Supplementary Material

Demographic, premorbid, and clinical characteristics of schizophrenia spectrum patients with high and low polygenic liability to the disorder

Margarita Alfimova *, Marina Gabaeva, Tatyana Lezheiko, Victoria Plakunova, Yulia Chaika, Vera Golimbet

***Corresponding author**: Dr. Margarita Alfimova; Mental Health Research Center, e-mail: m.alfimova@gmail.com

Content

Table S1. Definitions and coding of phenotypes and the number of patients included in the respective analysis	2
Table S2. Age distribution by group	4
Table S3. Phenotypic differences between the top and bottom deciles according to logistic regression	5
Table S4. Phenotypic differences between the top and bottom deciles according to logistic regression with sex as an additional covariate	9
Table S5. Results of the stepwise logistic regression to predict the top decile membership (vs bottom decile) based on statistically significant phenotypes from Table S3	11
Random Forest Analysis Notes	12
Table S6. Evaluation metrics	12
Table S7. Feature importance	13
Figure S1. ROC Curves Plot	14

Table S1. Definitions and coding of phenotypes and the number of patients included in the respective analysis (N)

Phenotype	Description and coding	N
Demographic characteristics		
Family history of schizophrenia spectrum and affective disorders or suicide attempts	1 - presence of a disorder of the schizophrenia and affective spectrum, as well as suicides in a first or second degree biological relative, 0 – absence.	156
Family history of any neuropsychiatric condition	1 - presence of any neuropsychiatric condition in a first or second degree biological relative (alcoholism, epilepsy, and parkinsonism were found in the present sample, besides schizophrenia and affective disorders); 0 – absence.	156
Sex	1 – female, 0 - male.	172
Number of siblings	1 - three or more children in the family, 0 - less than three children in the family.	135
Birth order	1 - firstborn, 0 – others.	135
Educational attainment (for patients ≥ 18 years)	codes from 0 to 8 according to the International Standard Classification of Education (ISCED-2011).	137
Marital history (for patients ≥ 18 years)	1 - ever been married or had a cohabitee, 0 - never had.	123
Current paid employment or education (for patients ≥ 18 years)	1 - current paid employment or training/education, 0 – absence.	132
Environmental risk factors		
Season of birth (SOB)	1 - birth in the winter months: December-February; 0 - in the others.	164
Urbanicity	1 - birth and early childhood in a big city (> 100,000); 2 - a small town, 3 - a village.	134
Obstetric complications (OC)	1 - presence of any of the following: preterm birth (<37 weeks), cesarean section, asphyxia/hypoxia, low birth weight, congenital malformations, cord complications, forceps or vacuum delivery, incubator or resuscitation, non-vertex presentation, birth injury, Rhesus incompatibility, twin birth, pre/eclampsia; 0 – absence.	142
Hypoxia/Asphyxia	1 - presence, 0 – absence.	140
Low birth weight	1 - presence, 0 – absence.	140
Adverse childhood experiences (ACE)	1 - presence of any of childhood maltreatment or dysfunctional household signs (physical or sexual abuse; peer victimization; foster care; parental mental illness; parental substance use disorder; parental suicidality, incarceration, or death; domestic violence); 0 – absence.	135
Premorbid somatic factors	1 - presence of any of the following: severe somatic or infectious diseases, surgery, traumatic brain injuries, 0 – absence.	117
Premorbid illicit drug use	1 - any report of use of marijuana or other drugs (did not include alcohol or smoking), 0 - data on the absence of the use.	93

Premorbid characteristics (antecedents)		
Premorbid neurologic signs and conditions	1 - presence of any of the following: enuresis, sleepwalking, seizures/episyndrome, tics, neurological follow-up for other problems (e.g., hydrocephalus), developmental delay, diagnoses of neurodevelopmental disorders (intellectual disability, autism spectrum disorders, attention deficit disorder with hyperactivity), 0 – absence.	121
Premorbid cognitive functioning	1 – high/good level (good academic performance, sufficient to enter a university), 2 - low level (below average academic performance with subsequent vocational training), 3 – very low level (obvious learning difficulties, such as duplication of classes), 4 - intellectual disability requiring special training.	145
Premorbid childhood social withdrawal	0 - no features, 1 – mild (shyness, isolation, few or no friends), 2 - severe communication problems, including pronounced autistic features.	93
Premorbid adolescent asocial behavior (for patients with age at illness onset > 12 years)	0 - absence, 1 – mild (decrease in academic performance, conflicts at school); 2 -severe (a member of an antisocial group, the use of psychoactive substances or alcohol).	92
Clinical characteristics		
ICD-10 diagnosis	Schizophrenia, schizoaffective disorder, schizotypal disorder.	172
Age at illness onset	Age of significant changes in behavior that may indicate the onset of the disease.	170
Age at illness onset (categorical)	COS – childhood schizophrenia (<13 years), EOS – early onset schizophrenia (13-17 years), AOS – adult-onset schizophrenia (at 18 years or older).	172
Age at first hospitalization	Age of the first admission to a psychiatric hospital.	166
Lifetime ICD symptoms (for patients > 12 years of age)	1 - the presence at any time in the course of the disease of one of the 7 main symptoms required for a diagnosis of schizophrenia according to ICD-10/11: delusions, hallucinations, disorganized thinking, experiences of passivity and control, negative symptoms, grossly disorganized behavior, psychomotor symptoms.	149
Psychomotor (catatonia) domains	1 - presence, 0 – absence.<br>Hyperkinetic: agitation/excitement, impulsivity, combativeness.<br>Hypokinetic: stupor, negativism, mutism.<br>Abnormal: grimacing, echolalia/ echopraxia, waxy flexibility, stereotypy, catalepsy, mannerism, posturing.	144-148
Suicidality	0 - absent, 1 – suicidal ideation, 2 - self-harm, 3 - attempt, 4 - multiple attempts.	138
Lifetime substance misuse (for patients > 12 years)	1 - presence, 0 – absence.	117

Table S2. Age distribution by group

Bottom Decile				Top Decile			
**Age**	**Frequency**	**Percent**	**Cumulative percent**	**Age**	**Frequency**	**Percent**	**Cumulative percent**
5	1	1.163	1.163	7	2	2.326	2.326
6	1	1.163	2.326	11	2	2.326	4.651
7	1	1.163	3.488	12	1	1.163	5.814
9	1	1.163	4.651	14	1	1.163	6.977
10	1	1.163	5.814	15	2	2.326	9.302
12	1	1.163	6.977	16	2	2.326	11.628
13	3	3.488	10.465	17	2	2.326	13.953
14	1	1.163	11.628	18	11	12.791	26.744
15	1	1.163	12.791	19	6	6.977	33.721
16	1	1.163	13.953	20	3	3.488	37.209
17	2	2.326	16.279	21	6	6.977	44.186
18	4	4.651	20.930	22	2	2.326	46.512
19	5	5.814	26.744	23	1	1.163	47.674
20	8	9.302	36.047	24	3	3.488	51.163
21	3	3.488	39.535	26	3	3.488	54.651
22	4	4.651	44.186	27	3	3.488	58.140
23	4	4.651	48.837	28	2	2.326	60.465
24	4	4.651	53.488	29	2	2.326	62.791
25	3	3.488	56.977	30	2	2.326	65.116
26	5	5.814	62.791	31	2	2.326	67.442
27	1	1.163	63.953	32	4	4.651	72.093
28	4	4.651	68.605	33	3	3.488	75.581
29	1	1.163	69.767	34	2	2.326	77.907
30	1	1.163	70.930	35	2	2.326	80.233
31	2	2.326	73.256	36	1	1.163	81.395
32	2	2.326	75.581	37	3	3.488	84.884
33	1	1.163	76.744	38	2	2.326	87.209
35	3	3.488	80.233	41	1	1.163	88.372
37	2	2.326	82.558	42	1	1.163	89.535
39	2	2.326	84.884	45	1	1.163	90.698
40	1	1.163	86.047	46	1	1.163	91.860
42	2	2.326	88.372	49	1	1.163	93.023
44	2	2.326	90.698	51	2	2.326	95.349
47	1	1.163	91.860	55	1	1.163	96.512
53	1	1.163	93.023	56	1	1.163	97.674
55	1	1.163	94.186	60	1	1.163	98.837
56	2	2.326	96.512	62	1	1.163	100.000
59	1	1.163	97.674	Missing	0	0.000	
64	1	1.163	98.837	Total	86	100.000	
66	1	1.163	100.000				
Missing	0	0.000					
Total	86	100.000					

Table S3. Phenotypic differences between the Top and Bottom deciles according to logistic regression

Phenotype	% of patients with the phenotype Top	Bottom	Logistic regression parameters (improvement compared to the null model[a])	OR, 95% CI to belong to the top decile	OR*, bca 95% CI to belong to the top decile
Family history of schizophrenic, affective disorders or suicide attempts	46.1	28.8	$\chi2$=5.27; p=0.022 (**p_{BH}=0.081**); R^2_N=0.05	OR=2.33; CI: 1.12-4.84; p=0.024	OR=2.61; CI: 0.96-4.63
Family history of any neuropsychiatric condition	67.1	41.3	$\chi2$=9.33; p=0.002 (**p_{BH}=0.037**); R^2_N=0.08	OR=3.02; CI: 1.46-6.25; p=0.003	OR=3.09; CI: 1.25-6.88
Female sex	60.5	37.2	$\chi2$=7.75; p=0.005 (**p_{BH}=0.046**); R^2_N=0.06	OR=2.95; CI: 1.35-6.41; p=0.006	OR=3.11; CI: 1.31-6.75
Number of siblings	10.6	11.6	$\chi2$=0.001; p=0.974 (p_{BH}=0.977); R^2_N<0.01	OR=0.98; CI: 0.31-3.13; p=0.974	OR=1.04; CI: 0.22-3.24
Birth order	76.6	66.2	$\chi2$=1.57; p=0.210 (p_{BH}=0.457); R^2_N=0.02	OR=1.69; CI: 0.74-3.85; p=0.213	OR=1.83; CI: 0.53-3.76
Educational attainment (median, 25%-75%)	4(2-6)	5(3-7)	$\chi2$=0.75; p=0.387 (p_{BH}=0.651); R^2_N=0.01	OR=0.92; CI: 0.75-1.12; p=0.388	OR=0.92; CI: 0.72-1.17
Marital history	24.6	31.0	$\chi2$=0.32; p=0.575 (p_{BH}=0.760); R^2_N<0.01	OR=0.75; CI: 0.27-2.08; p=0.575	OR=0.71; CI: 0.25-2.51
Current paid employment or education	28.4	36.9	$\chi2$=0.07; p=0.796 (p_{BH}=0.950); R^2_N<0.01	OR=0.90; CI: 0.39-2.06; p=0.796	OR=0.94; CI: 0.33-2.00
Season of Birth (SOB)	15.9	22.0	$\chi2$=0.23; p=0.628 (p_{BH}=0.801); R^2_N<0.01	OR=0.81; CI: 0.34-1.92; p=0.629	OR=0.77; CI: 0.31-2.58
Urbanicity[b] (city > 100000)	94.3; 4.7; 0	90.0; 4.3; 5.7	-	-	-
Obstetric Complications (OC)	37.3	41.3	$\chi2$=0.01; p=0.942 (p_{BH}=0.977); R^2_N<0.01	OR=0.97; CI: 0.45-2.10; p=0.942	OR=1.01; CI: 0.38-2.20
Hypoxia/Asphyxia	15.2	14.9	$\chi2$=0.34; p=0.558 (p_{BH}=0.760); R^2_N<0.01	OR=1.35; CI: 0.49-3.72; p=0.558	OR=1.38; CI: 0.41-4.79
Low birth weight[b]	9.1	8.1	-	-	-

Adverse childhood experiences (ACE)	60.3	44.4	$\chi2$=2.45; p=0.118 (p_{BH}=0.306); R^2_N=0.03	OR=1.80; CI: 0.86-3.78; p=0.120	OR=1.92; CI: 0.73-3.74
Premorbid somatic factors	8.5	13.8	$\chi2$=0.07; p=0.795 (p_{BH}=0.950); R^2_N<0.01	OR=0.85; CI: 0.24-2.99; p=0.795	OR=0.84; CI: 0.17-3.84
Premorbid illicit drug use	8.3	20.0	$\chi2$=2.36; p=0.124 (p_{BH}=0.306); R^2_N=0.04	OR=0.36; CI: 0.09-1.39; p=0.137	OR=0.35; CI: 0.03-1.56
Any environmental risk factor	75.3	85.9	$\chi2$=2.71; p=0.100 (p_{BH}=0.285); R^2_N=0.03	OR=0.47; CI: 0.19-1.17; p=0.104	OR=0.45; CI: 0.17-1.43
Premorbid neurological signs and conditions	16.7	31.1	$\chi2$=6.79; p=0.009 (**p_{BH}=0.067**); R^2_N=0.08	OR=0.27; CI: 0.10-0.76; p=0.013	OR=0.25; CI: 0.08-0.82
Premorbid cognitive functioning: high; low; very low; intellectual disability (ID)	42.0; 44.9; 10.2; 2.9	53.9; 22.4; 9.2; 14.5	$\chi2$=11.22; p=0.011 (**p_{BH}=0.068**); R^2_N=0.10	low vs high: OR=2.31; CI: 1.01-5.27; p=0.048; very low vs high: OR=1.27; CI: 0.37-4.36; p=0.708; ID vs high: OR=0.18; CI: 0.03-1.09; p=0.062	low vs high: OR=2.31; CI: 0.96-5.63; very low vs high: OR=1.29; CI: 0.24-6.04; ID vs high: OR=0.16; CI: 0.00-1.68
Premorbid childhood social withdrawal: absent; mild; severe	38.6; 56.8; 4.6	53.1; 26.5; 20.4	$\chi2$=14.46; p<0.001 (**p_{BH}<0.037**); R^2_N=0.21	mild vs absent: OR=3.57; CI: 1.25-10.21; p=0.018; severe vs absent: OR=0.17; CI: 0.03-1.07; p=0.059	mild vs absent: OR=4.17; CI: 0.98-12.40; severe vs absent: OR=0.15; CI: 0.00-4.69
Premorbid adolescent asocial behavior: absent; mild; severe	63.6; 20.5; 15.9	64.6; 12.5; 22.9	$\chi2$=2.60; p=0.273 (p_{BH}=0.561); R^2_N=0.04	mild vs absent: OR=1.86; CI: 0.45-7.65, p=0.389; severe vs absent: OR=0.48; CI: 0.13-1.74, p=0.266	mild vs absent: OR=1.93; CI: 0.38-16.42; severe vs absent: OR=0.43; CI: 0.15-2.93
ICD-10 diagnosis: F21; F25; F20	1.2; 12.8;	14.0; 8.1;	$\chi2$=8.37; p=0.015	F21 vs F20: OR=0.11; CI:	F21 vs F20: OR=0.08;

	86.0	77.9	(**p_{BH}=0.074**); R^2_N=0.07	0.01-0.87; p=0.037. F25 vs F20: OR=1.60; CI: 0.50-5.10; p=0.426	CI: 0.00-1.67; F25 vs F20: OR=1.79; CI: 0.32-5.15
Age at illness onset (years)	17.5± 6.4	17.6± 8.00	χ2=0.65; p=0.422 (p_{BH}=0.651); R^2_N=0.01	OR=1.02; CI: 0.98-1.08; p=0.423	OR=1.03; CI: 0.95-1.08
Age at first hospitalization	19.5± 7.4	20.4± 8.8	χ2=0.01; p=0.937 (p_{BH}=0.977); R^2_N<0.01	OR=1.00; CI: 0.95-1.06; p=0.937	OR=1.01; CI: 0.95-1.05
Age at illness onset: COS, EOS, AOS	12.8; 47.7; 39.5	17.4; 34.9; 47.7	χ2=1.90; p=0.386 (p_{BH}=0.651); R^2_N=0.02	COS vs AOS: OR=0.52; CI: 0.16-1.65; p=0.265; EOS vs AOS: OR=1.12; CI: 0.53-2.36; p=0.769	COS vs AOS: OR=0.48; CI: 0.14-2.02; EOS vs AOS: OR=1.12; CI: 0.46-2.52
Lifetime number of ICD symptoms (median, 25%-75%)	5 (3-5)	4 (2-5)	χ2=1.07; p=0.301 (p_{BH}=0.566); R^2_N=0.01	OR=1.13; CI: 0.90-1.41; p=0.304	OR=1.14; CI: 0.87-1.46
Lifetime delusions	96.1	76.4	χ2=5.84; p=0.016 (**p_{BH}=0.074**); R^2_N=0.06	OR=4.58; CI: 1.17-17.84; p=0.028	OR=5.91; CI: 1.08-21.51
Lifetime hallucinations	76.6	61.6	χ2=3.07; p=0.080 (p_{BH}=0.269); R^2_N=0.03	OR=2.05; CI: 0.91-4.62; p=0.082	OR=2.09; CI: 0.81-5.54
Lifetime disorganized thinking	76.6	67.7	χ2=0.64; p=0.425 (p_{BH}=0.651); R^2_N=0.01	OR=1.40; CI: 0.61-3.21; p=0.425	OR=1.41; CI: 0.52-3.67
Lifetime experiences of passivity and control	26.0	25.7	χ2=0.04; p=0.840 (p_{BH}=0.972); R^2_N<0.01	OR=0.92; CI: 0.39-2.16; p=0.840	OR=0.92; CI: 0.32-2.23
Lifetime negative symptoms	72.4	70.8	χ2=0.36; p=0.550 (p_{BH}=0.760); R^2_N<0.01	OR=0.77; CI: 0.33-1.80; p=0.551	OR=0.78; CI: 0.26-1.95
Lifetime grossly disorganized behavior	30.1	31.5	χ2=0.60; p=0.440 (p_{BH}=0.651); R^2_N=0.01	OR=0.71; CI: 0.30-1.70; p=0.442	OR=0.68; CI: 0.30-2.23
Lifetime psychomotor symptoms	61.0	47.2	χ2=1.75; p=0.186 (p_{BH}=0.430); R^2_N=0.02	OR=1.68; CI: 0.78-3.63; p=0.188	OR=1.70; CI: 0.63-4.16

Psychomotor - Hyperkinetic type	58.7	56.2	$\chi2<0.01$; p=0.977 (p_{BH}=0.977); $R^2_N<0.01$	OR=0.99; CI: 0.45-2.17; p=0.977	OR=1.03; CI: 0.38-2.25
Psychomotor – Hypokinetic type	15.1	9.9	$\chi2=2.72$; p=0.099 (p_{BH}=0.285); $R^2_N=0.03$	OR=2.61; CI: 0.82-8.35; p=0.106	OR=2.83; CI: 0.73-7.80
Psychomotor - Abnormal type	39.7	20.8	$\chi2=5.39$; p=0.020 (**p_{BH}=0.081**); $R^2_N=0.05$	OR=2.71; CI: 1.15-6.42; p=0.023	OR=3.01; CI: 1.06-6.95
Suicidality (median, 25%-75%)	0 (0-1)	0 (0-1)	$\chi2=0.03$; p=0.959 (p_{BH}=0.977); $R^2_N<0.01$	OR=0.99; CI: 0.74-1.33; p=0.959	OR=1.00; CI: 0.68-1.34
Lifetime substance misuse	28.8	37.9	$\chi2=1.05$; p=0.306 (p_{BH}=0.566); $R^2_N=0.01$	OR=0.65; CI: 0.28-1.50; p=0.308	OR=0.65; CI: 0.23-1.52
PANSS Positive factor (scores, M±SD)[c]	23.86± 5.96	20.21± 6.14	Wald Stat=8.98; p=0.003 (**p_{BH}=0.037**)	OR=1.12; CI: 1.04-1.21; P=0.003	OR=1.13; CI: 1.03-1.21

Notes. [a] Null model included age, two PCs for ancestry, and genotyping site. For lifetime ICD symptoms it additionally included illness duration.

[b]- phenotypes equal in more than 90% of the sample were not analyzed.

[c] - for symptoms severity a stepwise logistic regression was used, all six PANSS factors being analyzed as predictors simultaneously; the best model included the second ancestry PC, genotyping site, and the PANSS Positive factor. PANSS scores were available for 123 patients.

Abbreviations: AOS - adult illness onset, ≥18 years; EOS – early onset,13-17 years; COS – childhood schizophrenia, onset before 13 years.

Table S4. Phenotypic differences between the Top and Bottom deciles according to logistic regression with sex as an additional covariate

Phenotype	Logistic regression parameters	OR, 95% CI to belong to the top decile	OR*, bca 95% CI to belong to the top decile
Family history of schizophrenic and affective disorders or suicide attempts	$\chi2$=4.16; p=0.04 (p_{BH}=0.185); R^2_N=0.04	OR=2.18; CI: 1.02-4.66; p=0.044	OR=2.34; CI: 0.86-4.96
Family history of any neuropsychiatric condition	$\chi2$=8.15; p=0.004 (**p_{BH}=0.048**); R^2_N=0.07	OR=2.88; CI: 1.37-6.04; p=0.005	OR=3.26; CI: 0.98-5.69
Number of siblings	$\chi2$=0.01; p=0.930 (p_{BH}=0.957); R^2_N<0.01	OR=0.95; CI: 0.30-3.06; p=0.930	OR=1.04; CI: 0.19-3.00
Birth order	$\chi2$=3.66; p=0.056 (p_{BH}=0.202);R^2_N=0.04	OR=2.36; CI: 0.96-5.59; p=0.060	OR=2.43; CI: 0.86-6.59
Educational attainment	$\chi2$=0.59; p=0.442 (p_{BH}=0.758); R^2_N=0.01	OR=0.94; CI: 0.75-1.13; p=0.442	OR=0.94; CI: 0.72-1.13
Marital history	$\chi2$=0.81; p=0.368 (p_{BH}=0.664); R^2_N=0.01	OR=0.63; CI: 0.21-1.79; p=0.370	OR=0.56; CI: 0.19-2.60
Current paid employment or education	$\chi2$=0.23; p=0.633 (p_{BH}=0.764); R^2_N<0.01	OR=0.81; CI: 0.35-1.91; p=0.633	OR=0.78; CI: 0.34-2.44
Season of Birth	$\chi2$=0.24; p=0.628; (p_{BH}=0.764); R^2_N<0.01	OR=0.80; CI: 0.33-1.95; p=0.628	OR=0.79; CI: 0.22-2.43
Obstetric Complications	$\chi2$=0.02; p=0.902 (p_{BH}=0.955); R^2_N<0.01	OR=1.05; CI: 0.48-2.33; p=0.902	OR=1.13; CI: 0.33-2.44
Hypoxia/Asphyxia	$\chi2$=1.19; p=0.275 (p_{BH}=0.582); R^2_N=0.01	OR=1.79; CI: 0.63-5.13; p=0.276	OR=2.11; CI: 0.38-4.96
Adverse childhood experiences	$\chi2$=2.15; p=0.143 (p_{BH}=0.396); R^2_N=0.02	OR=1.75; CI: 0.83-3.73; p=0.144	OR=1.80; CI: 0.73-3.85
Premorbid somatic factors	$\chi2$=0.24; p=0.624 (p_{BH}=0.764); R^2_N<0.01	OR=0.73; CI: 0.20-2.62; p=0.625	OR=0.73; CI: 0.16-3.12
Premorbid illicit drug use	$\chi2$=1.81; p=0.179; (p_{BH}=0.460); R^2_N=0.03	OR=0.40; CI: 0.10-1.59; p=0.191	OR=0.37; CI: 0.05-2.15
Any environmental risk factor	$\chi2$=2.61; p=0.106 (p_{BH}=0.318); R^2_N=0.03	OR=0.46; CI: 0.18-1.20; p=0.111	OR=0.44; CI: 0.16-1.34
Premorbid neurological signs and conditions	$\chi2$=5.92; p=0.015 (**p_{BH}=0.096**); R^2_N=0.07	OR=0.29; CI: 0.10-0.82; p=0.020	OR=0.27; CI: 0.09-1.04
Premorbid cognitive functioning: high; low; very low; intellectual disability (ID)	$\chi2$=10.29; p=0.016 (**p_{BH}=0.096**); R^2_N=0.10	low vs high: OR=2.12; CI: 0.90-4.96; p=0.085; very low vs high: OR=1.23; CI: 0.35-4.33; p=0.750; ID vs high: OR=0.16; CI: 0.03-1.04; p=0.055	low vs high: OR=2.15; CI: 0.82-5.20; very low vs high: OR=1.23; CI: 0.29-5.06; ID vs high: OR=0.14; CI: 0.00-2.01
Premorbid childhood social withdrawal (absent; mild; severe)	$\chi2$=12.45; p=0.002 (**p_{BH}=0.048**); R^2_N=0.18	mild vs absent: OR=3.49; CI: 1.20-10.13; p=0.021; severe vs absent: OR=0.19; CI: 0.03-1.20; p=0.078	mild vs absent: OR=4.16; CI: 0.58-13.37; severe vs absent: OR=0.15; CI: 0.00-7.45

Premorbid adolescent asocial behavior	$\chi 2$=1.10; p=0.577 (p_{BH}=0.764); R^2_N=0.02	mild vs absent: OR=1.63; CI: 0.38-7.02; p=0.509; severe vs absent: OR=0.63; CI: 0.16-2.52; p=0.513	mild vs absent: OR=1.57; CI: 0.30-1252306; severe vs absent: OR=0.62; CI: 0.12-2.85
ICD-10 diagnosis (F21; F25; F20)	$\chi 2$=5.96; p=0.051 (p_{BH}=0.202); R^2_N=0.05	F21vs F20: OR=0.15; CI: 0.02-1.25; p=0.080; F25 vs F20: OR=1.68; CI: 0.51-5.53; p=0.396;	F21 vs F20: OR=0.14; CI: 0.00-1.38; p=0.080; F25 vs F20: OR=1.88; CI: 0.47-5.79
Age at illness onset (years)	$\chi 2$=0.38; p=0.535 (p_{BH}=0.764); R^2_N=0.01	OR=1.02; CI: 0.96-1.08; p=0.535	OR=1.02; CI: 0.96-1.09
Age at first hospitalization	$\chi 2$=0.02; p=0.896 (p_{BH}=0.955); R^2_N<0.01	OR=1.00; CI: 0.94-1.05; p=0.896	OR=1.00; CI: 0.93-1.05
Age at illness onset (COS, EOS, AOS)	$\chi 2$=1.52; p=0.468 (p_{BH}=0.764); R^2_N=0.01	COS vs AOS: OR=0.56; CI: 0.17-1.85; p=0.340; EOS vs AOS: OR=1.13; CI: 0.53-2.43; p=0.757	COS vs AOS: OR=0.52; CI: 0.16-2.49; EOS vs AOS: OR=1.16; CI: 0.52-2.46
Lifetime number of ICD symptoms	$\chi 2$=0.40; p=0.526 (p_{BH}=0.764); R^2_N<0.01	OR=1.08; CI: 0.85-1.36; p=0.526	OR=1.07; CI: 0.84-1.43
Lifetime delusions	$\chi 2$=4.66; p=0.031 (p_{BH}=0.159); R^2_N=0.05	OR=3.99; CI: 1.01-15.70; p=0.048	OR=4.81; CI: 0.96-19.15
Lifetime hallucinations	$\chi 2$=1.38; p=0.241 (p_{BH}=0.542); R^2_N=0.01	OR=1.66; CI: 0.71-3.89; p=0.242	OR=1.67; CI: 0.61-4.28
Lifetime disorganized thinking	$\chi 2$=0.22; p=0.637 (p_{BH}=0.764); R^2_N<0.01	OR=1.23; CI: 0.52-2.89; p=0.637	OR=1.20; CI: 0.46-3.69
Lifetime experiences of passivity and control	$\chi 2$=0.36; p=0.546 (p_{BH}=0.764); R^2_N<0.01	OR=0.76; CI: 0.31-1.86; p=0.547	OR=0.75; CI: 0.29-2.40
Lifetime negative symptoms	$\chi 2$=0.17; p=0.680 (p_{BH}=0.790); R^2_N<0.01	OR=0.83; CI: 0.35-1.98; p=0.681	OR=0.85; CI: 0.29-2.18
Lifetime grossly disorganized behavior	$\chi 2$=0.81; p=0.369 (p_{BH}=0.664); R^2_N=0.01	OR=0.67; CI: 0.27-1.63; p=0.373	OR=0.61; CI: 0.24-2.19
Lifetime psychomotor symptoms	$\chi 2$=1.69; p=0.194 (p_{BH}=0.466); R^2_N=0.02	OR=1.67; CI: 0.77-3.65; p=0.195	OR=1.71; CI: 0.69-3.98
Psychomotor -Hyperkinetic type	$\chi 2$<0.01; p=0.972 (p_{BH}=0.972); R^2_N<0.01	OR=1.01; CI: 0.46-2.25; p=0.972	OR=0.99; CI: 0.43-2.91
Psychomotor – Hypokinetic type	$\chi 2$=3.15; p=0.076 (p_{BH}=0.249); R^2_N=0.03	OR=2.91; CI: 0.87-9.69; p=0.083	OR=3.12; CI: 0.75-10.61
Psychomotor - Abnormal type	$\chi 2$=6.00; p=0.014 (**p_{BH}=0.096**); R^2_N=0.06	OR=2.98; CI: 1.21-7.30; p=0.017	OR=3.40; CI: 1.10-7.28
Suicidality	$\chi 2$=0.08; p=0.777 (p_{BH}=0.874); R^2_N<0.01	OR=0.96; CI: 0.71-1.29; p=0.777	OR=0.97; CI: 0.65-1.29
Lifetime substance misuse	$\chi 2$=0.93; p=0.334 (p_{BH}=0.664); R^2_N=0.01	OR=0.66; CI: 0.28-1.55; p=0.336	OR=0.66; CI: 0.25-1.70
PANSS Positive factor	Wald Stat=8.98; p=0.003 (**p_{BH}=0.048**)	OR=1.21; CI: 1.04-1.21	OR=1.13; CI: 1.04-1.20

Table S5. Results of the stepwise logistic regression to predict the top decile membership (vs bottom decile) based on statistically significant phenotypes from Table S3

Predictor	OR, 95% CI, to belong to the top decile	Wald Statistic	p-value
Intercept	OR=0.01; CI: 0.00-0.22	8.92	0.003
PANSS Positive factor	OR=1.16; CI: 1.04-1.30	6.89	0.009
Family history of any neuropsychiatric condition	OR=29.31; CI: 2.38-361.54	6.95	0.008
Premorbid childhood social withdrawal, mild vs absent	OR=4.03; CI: 1.11-14.67	4.46	0.034
Premorbid childhood social withdrawal, severe vs absent	OR=0.19; CI: 0.01-3.78	1.20	0.273
Family history of schizophrenia and affective disorders or suicide attempts	OR=0.91; CI: 0.01-1.13	3.49	0.062

Note. Model parameters: Sample n=67; Variables from the initial pool of predictors not included in the final model: age, sex, PC1, PC2, genotyping site, illness duration, premorbid neurological signs and conditions, premorbid cognitive functioning, ICD-10 diagnosis, lifetime delusions, psychomotor symptoms – abnormal type. Nagelkerke R^2=0.49.

Random Forest Analysis Notes

Methods
A random forest analysis was performed to predict BD and TD status using 36 feature variables (see Table S1, with PANSS scores being excluded). Individuals with missing data were not included and the sample consisted of 54 patients with complete data. Random forest classification analysis was performed using JASP 0.16, which in turn uses the R packages "randomForest" and ROCR. Default settings were used except that we chose to randomly select 30% of the data to obtain the prediction error. The default settings were as follows: 20% sample for validation data, 50% training data used per tree, and the maximum number of possible decision trees was 100.

Results
The model parameters were as follows: 31 trees, six features per split, training sample -35, validation sample – 9, test sample – 10; validation accuracy – 0.556, test accuracy – 0.500, out-of-bag accuracy – 0.684. Evaluation metrics, parameters of feature importance and ROC are presented in Tables S6 and S7 and Fig. S1.

Table S6. Evaluation metrics

Metrics	Bottom	Top	Average/Total
Support	6	4	10
Accuracy	0.500	0.500	0.500
Precision (Positive Predictive Value)	0.667	0.429	0.571
Recall (True Positive Rate)	0.333	0.750	0.500
False Positive Rate	0.250	0.667	0.458
False Discovery Rate	0.333	0.571	0.452
F1 Score	0.444	0.545	0.485
Matthews Correlation Coefficient	0.089	0.089	0.089
Area Under Curve (AUC)	0.542	0.625	0.583
Negative Predictive Value	0.429	0.667	0.548
True Negative Rate	0.750	0.333	0.542
False Negative Rate	0.667	0.250	0.458
False Omission Rate	0.571	0.333	0.452
Threat Score	0.333	0.333	0.333
Statistical Parity	0.300	0.700	1.000

Table S7. Feature importance

Feature	Mean decrease in accuracy	Total increase in node purity
Number of lifetime ICD-10 symptoms	0.021	0.035
Family history of any neuropsychiatric condition	-0.010	0.014
Premorbid cognitive functioning	0.010	0.014
Lifetime hallucinations	0.026	0.013
Lifetime psychomotor symptoms	-0.007	0.012
Premorbid neurological signs and conditions	0.003	0.011
Lifetime psychomotor symptoms: Abnormal Type	0.010	0.005
Premorbid childhood social withdrawal	0.039	0.004
Grossly disorganized behavior	0.000	0.004
SOB	0.006	0.003
OC	0.000	0.003
Premorbid adolescent asocial behavior	-0.007	0.002
Marital history	0.000	0.002
Sex	-0.003	0.002
ACE	0.000	6.517×10^{-4}
Age at illness onset (categorical)	0.000	0.000
Hypoxia	0.000	0.000
Premorbid somatic factors	0.000	0.000
Lifetime substance misuse	-0.004	0.000
Any environmental risk factor	0.003	0.000
Number of siblings	0.000	0.000
Current paid employment or education	0.000	0.000
Lifetime psychomotor symptoms: Hyperkinetic Type	0.000	0.000
Lifetime psychomotor symptoms: Hypokinetic Type	0.003	0.000
Lifetime delusions	0.000	0.000
Lifetime negative symptoms	0.003	0.000
Educational attainment	-0.007	-5.376×10^{-4}
Age at illness onset	0.002	-0.003
Premorbid illicit drug use	-0.004	-0.003
Lifetime disorganized thinking	-0.004	-0.004
Suicidality	-0.004	-0.005
Lifetime experiences of passivity and control	0.000	-0.007
Age at first hospitalization	-0.003	-0.008
ICD-10 diagnosis	0.009	-0.009
Family history of schizophrenic and affective disorders or suicide attempts	0.005	-0.012
Birth order	-0.007	-0.020

Figure S1. ROC Curves Plot

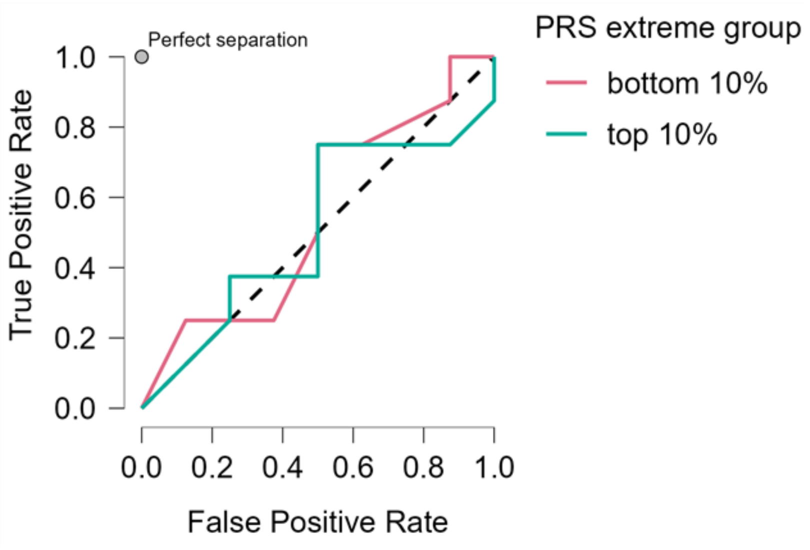

Supplement: Supplementary file 1 [file diseases-13-00066-s001.zip › diseases-3476224-supplementary.pdf]
